# ESport programs in high school: what’s at play?

**DOI:** 10.3389/fpsyt.2024.1306450

**Published:** 2024-01-26

**Authors:** Antoine Lemay, Magali Dufour, Mathieu Goyette, Djamal Berbiche

**Affiliations:** ^1^ Département de Psychologie, Université du Québec à Montréal, Montreal, QC, Canada; ^2^ Département de Sexologie, Université du Québec à Montréal, Montréal, QC, Canada; ^3^ Départment des Sciences de la Santé Communautaire, Université de Sherbrooke, Longueuil, QC, Canada

**Keywords:** adolescent, gaming, eSports, gaming disorder, mental health, education, prevention

## Abstract

**Background:**

A growing number of high schools in Canada offer eSports (ES) in their facilities, which raises concerns regarding this activity’s potential health risks for adolescents.

**Methods:**

The aim of this study is to describe the characteristics of 67 adolescent ES players (ESp) and to compare them to 109 recreational gamers in their high school (nESp). The two groups were compared on (1) sociodemographic and academic characteristics; (2) online and offline activities; (3) psychological characteristics.

**Results:**

Results show that ESp spend more time on online activities and report a higher proportion of problematic gaming compared to the nESp group. ESp report more often that gaming has positive consequences on their physical health and report more often negative consequences on their education compared to the nESp group.

**Conclusion:**

These results underscore the importance of screening gaming problems among adolescent ES players. Targeted prevention should be carried out with these teenagers and in order to be adapted, prevention efforts should consider both, the positive and negative consequences that ESp experience from gaming.

## 1 Introduction

The arrival of new technologies in all sectors of our lives has raised many questions about the effects of this integration in our daily lives. The educational sector is no exception to the challenges associated with technological evolution. For example, many Canadian provinces have developed action plans for the integration of digital technology in education (1–3). Today, 97% of high schools have an Internet connection in all classrooms and 74% of schools have a policy on the use of mobile devices (4). Equipped with these new technologies, schools have a dual mandate. On one hand, they must help young people develop digital skills, and on the other, they must do so within a safe framework (5). While the integration of technology is a vehicle for increasing student motivation and commitment, teachers, parents, and even UNESCO are concerned about the harmful effects of these tools (5, 6). Gamification of the educational environment – or using gaming components in school contexts - is a prime example of the controversy surrounding the integration of technology into schools. If gamification of academic content can support student learning (7), what about new video game activities as a sport-study program or as an extracurricular activity? Do video games have a place in high schools? This study will look specifically at the integration of eSport in high schools.

ESport (ES) is a recent phenomenon that differs from traditional recreational gaming. ES can be defined as a form of competitive gaming where structured competitions and tournaments are organized by various leagues in front of an audience (8, 9). Hence, considering its competitive nature, not all video games allow ES to take place as it requires games that facilitate confrontations between players, such as *League of Legends*, *Dota 2*, *Fortnite*, *Rocket League* or *Counter Strike*. Another key distinction between ES and traditional gaming is the important number of hours that ES players will spend practicing and developing strategies for competitions.

In recent years, the number of ES players is continually increasing and ES has become a worldwide phenomenon. ES is so popular that more than 500 million people watch ES every year and its associated annual market is estimated at US $1.3 billion (10, 11). In the last year, 3 800 Canadians registered in over 8 250 ES tournaments worldwide and earned a total of nearly US $42 million (12). ES is now so popular that doing it professionally is now an option for many gamers (12).

ES is well-established in higher education settings, thus encouraging video game competitions among universities (13–15). Recently, high schools have also started to include ES in curricular or extracurricular activities (16, 17). In Quebec (Canada), at least four high schools have introduced ES in their curriculum and over 10 other high schools have an extracurricular ES program (18, 19). Integrating ES into high schools has been the subject of much debate. On one hand, some suggests that offering ES in high schools can help supervise adolescents’ gaming activities and encourage their personal growth (20, 21). On the other hand, school counselors and parents are concerned about the health risks that could ensue from this activity – namely an increase in gaming disorder among adolescents (22, 23). In 2019, the concerns associated with ES in schools were such that some members of Parliament called for a ban on the integration of ES in high schools and a political debate was held in the *Assemblée Nationale* on these issues (24, 25). In order to address the concerns surrounding ES, it is important to have an accurate understanding of this practice and the risks and consequences associated with it.

While knowledge about adults ES players is increasing, little is known about adolescent players. Four studies have explored the profile of adolescent ES players (26–28), and to our knowledge, only one study documented ES programs in high school context (29). Martynenko & al (26) conducted a pilot study on 32 adolescents aged 13 to 17 years presenting an unspecified handicap in order to develop a psychosocial intervention program. However, the size and specific nature of the sample offer limited knowledge as in term of the profile of adolescent ES players from the “general population”. Kocadağ (27, 28) conducted two studies on adolescents’ psychological well-being and surveyed 320 (age range: 15–27) and 368 (age range: 15–25) young participants, respectively. Results show that gaming daily, especially more than six hours, is associated with lower psychological well-being, and that having an interest in an ES career predicts having a low psychological well-being (27, 28). Trotter & al. (29) conducted a study to compare 89 adolescents enrolled in an ES program to 99 adolescents from a matched control group on self-regulation, growth mindset (belief that one’s talents can be developed through hard work), positive youth development, physical activity and perceived general health. This study shows that the two groups are very similar, with the exception of a higher level of physical activity in the control group. Although interesting, these studies lack detailed information on participants’ profile, leisure and gaming time, and many other psychological variables (e.g., problematic gaming, psychological distress, and self-esteem) known to be risk factors for behavioral addictions and video game disorder (30). Consequently, due to the scarcity of the research on adolescents, documentation on adult ES players will be presented to gain insight on adolescent ES players’ profiles.

Recent years have seen the development of studies on adult ES players, particularly professionals. However, recent literature reviews show that knowledge about psychological characteristics remains limited (31, 32). To date, studies have focused mainly on advertisement, revenues, and regulations of ES (32). A significant number of articles have also looked at the professionalization, the skill development and ES spectatorship (31). While interesting, this literature does little to advance our understanding of adolescents in a school environment who take part in ES during their studies.

The first studies documenting the characteristics of adults ES players show that for the majority, they are young men aged between 19 and 25 (33–35), single (33) and highly educated (33–35). These players devote a great deal of time to ES. In fact, these players spend five to 10 hours daily developing ES related skills (36–38). In addition to the time they spend on ES, they spend between 25 to 30 hours weekly on recreational gaming (35, 38–40). As a result, adult ES players could easily spend between 60 to 80 hours weekly on gaming activities.

Different motivations are associated with ES in adults. Extrinsic motivations such as competition, socialization and the possibility to escape problems, and intrinsic motivations such as fun, the need for diversion (e.g., new experiences) and affiliation (e.g., belonging) are often reported for ES (31, 33, 41). A study on ES motivations, conducted on 162 adult gamers including 108 ES players, showed that, in comparison to recreational players, ES players aim to satisfy specific life goals (42). More specifically, ES players are motivated by satisfying their need for diversion and affiliation compared to recreational gamers (42). Overall, adults are motivated in ES for a variety of reasons. Considering the lack of documentation on ES motivations in adolescents, little is known about their motivation towards this activity. Hence, it is difficult to know how extrinsically or intrinsically motivated adolescents are towards ES, and how they may differ from adult players.

The effects of ES on physical health and, to a lesser extent mental health, have also been documented. Regarding physical health, ES is associated with a sedentary lifestyle, postural injuries, ocular fatigue, and sleep problems (37, 43–45). In terms of cognitive health, studies have shown that ES could have positive effects on players’ executive functions, hand-eye coordination, problem solving skills, reaction time, and working memory (37, 44, 45). The most important benefit associated with ES appears to be socialization (37, 44, 45). Freeman & Wohn (46) highlighted the importance of the social aspects of ES in players’ lives. Considering the important amount of time players spend with their team, they can develop close relationships with them, which can become a prime source of support when facing challenges (46).

Conversely, ES is also associated with psychosocial consequences – notably an increased risk in the development of gaming disorder (31, 33, 40, 41, 47). Since 2019, gaming disorder has been officially recognized as a mental health disorder by the World Health Organization and can be defined as: *“a pattern of gaming behavior (“digital-gaming” or “video-gaming”) characterized by impaired control over gaming, increasing priority given to gaming over other activities to the extent that gaming takes precedence over other interests and daily activities, and continuation or escalation of gaming despite the occurrence of negative consequences”* (48). Worldwide, 4.6% of adolescents will develop gaming disorder, 6.8% of males and 1.3% of females (49). Known risk factors for developing gaming disorder in adolescence include: being a male, gaming often and for many hours, experiencing family, social (e.g, being isolated, lacking social skills) or school difficulties (50–52). Other risk factors also include being more impulsive, anxious or depressed, presenting a low self-esteem and a lower life satisfaction (50–52). The few studies that have investigated this disorder among adult ES players report that they have more problematic gaming than recreational gamers, possibly ranging from 10 to 20% (33, 40, 41, 53). Since no studies have been conducted on adolescent ES players, it is difficult to conclude if they are also more at risk of developing gaming disorder.

To summarize the available documentation on ES players, most of the initial data are from adults and highlight potential positive and negative consequences of ES. However, these studies do not allow us a better understanding of adolescent ES players’ profiles and risk factors associated with developing gaming disorder. As schools are responsible for ensuring the safety of the activities offered to their students, it’s important to take a closer look at the profile of young ES players and the known risk factors for developing gaming disorder. The objective of this exploratory comparative study is to compare high school students that participates in an extracurricular ES program to their recreational gamer peers, controlling for (1) sociodemographic and academic characteristics; (2) offline and online activities (ES time, gaming time, perceived positive and negative consequences of gaming); and (3) psychological characteristics (problematic gaming, psychological distress, self-esteem, impulsivity, gaming motivations). This study design is relevant considering the presence of concerns surrounding ES among adolescents in school contexts, and the limited data available from this population.

## 2 Materials and methods

### 2.1 Context

This exploratory comparative study was carried out in 2020 in a high school (Québec, Canada) offering an extra-curricular ES program. This ES program allowed students to practice ES during lunchtime and offered two class periods weekly to participate in a competitive gaming league. The ES players in this program had parental authorization, had to maintain their academic results, participate in an extra weekly hour of sport, and attend a monthly prevention workshop on healthy lifestyle habits.

Since ES was only offered to students enrolled in 11^th^ and 12^th^ grade, this study used a convenience sample of adolescent high school students enrolled in 11^th^ and 12^th^ grade that reported gaming. All parents were informed by letter of the study and allowed the recruitment of their adolescent. In total, 11 classrooms were visited, and all adolescents were invited to participate in the study. To be eligible for the study, adolescents needed to speak French or English and report gaming or doing ES. Adolescents were asked to complete a 50-minute questionnaire on *LimeSurvey*. Two $25 gift certificates were drawn in each class to compensate the participants for their time.

### 2.2 Participants

In total, 219 adolescents participated in the study. However, ten participants were excluded for various reasons (e.g., uncompleted questionnaire, unclear answers, or maximum score on each scale). In order to be considered an ES player, participants were asked if they have done ES in the past 12 months. All participants that answered ‘yes’ were enrolled in the extracurricular ES program offered by the school, and thus formed the group ES player (ESp). One ESp and seven non-eSport players (nESp) were excluded since they reported spending no time gaming. Because of the very low number of girls in the ESp group (*n* = 1), only boys were considered in the study. The final sample is composed of 176 adolescent gamers enrolled in high school (M_age_ = 16.4) separated into two groups: ESp (*n* = 67) and nESp (*n* = 109).

### 2.3 Instruments

#### 2.3.1 Sociodemographic characteristics

A sociodemographic questionnaire composed of 12 questions was administered to document age, ethnic origin, family composition, relational status, employment status, average grades and average time spent studying per week.

#### 2.3.2 Experience in ES

The number of years of experience in ES and their desire to play professionally in the future were documented. The answers to the question about desire to continue ES were the following: (1) no, (2) yes, amateur, (3) yes, semi-professional, (4) yes, professional. The variable was coded “No” = 1-2 and “Yes” = 3-4.

#### 2.3.3 Offline and online activities

For all participants, the number of hours spent on offline and online activities (e.g., gaming, ES, social medias, *YouTube*, streaming) were documented. To reduce the effect of extreme scores, maximum time for leisure activities was capped at 50 hours per week. Total time spent on online activities was calculated by adding the time spent on: (1) gaming, (2) ES, and (3) other online leisure activities.

#### 2.3.4 Gaming-related expenses

Annual gaming-related expenses were documented through questions about the amount of money spent on the purchase of equipment or video games, memberships, skins (def: non-essential item, purely aesthetic, which changes the appearance of the character in the video game) and loot boxes (def: items in video games that are bought with real-world money and that contain randomized contents whose value is uncertain). To reduce the effect of extreme scores, the amounts spent were capped at three standard deviations from the mean. The total amounts participants spent were calculated by adding all expenses reported.

#### 2.3.5 Gaming motivations

Gaming motivations were assessed using the Gaming Motivation Scale (GAMS; 54). The GAMS is an 18-item questionnaire designed to assess gaming motivations. Items are rated on a Likert scale ranging from 1 (do not agree at all) to 7 (very strongly agree). The GAMS is divided into six subscales (intrinsic, integrated, identified, introjected, external, amotivation) forming a continuum between intrinsic and extrinsic motivations, or amotivation. All subscales present a Cronbach’s α coefficient superior to 0.75.

#### 2.3.6 Perceived positive and negative consequences of gaming

Perceived positive and negative consequences were evaluated using two questionnaires with 12 questions each, rated along a Likert scale ranging from 0 (not at all) to 10 (extremely; 55). The first questionnaire documented the positive consequences and the second recorded the negative consequences associated with gaming in the past year related to various spheres of life (work/study, social, psychological health). Scores associated with each indicator were dichotomized to compare “not at all/somewhat” (0-3) to “moderately/extremely” (4-10).

#### 2.3.7 Problematic gaming

Problematic gaming was assessed with a modified version for gaming of the Compulsive Internet Use Scale – CIUS-14 (56). The French version of the CIUS-14 was designed and validated to assess problematic Internet use in French speaking population and presents a Cronbach’s α coefficient of 0.90 (56, 57). The questionnaire uses a Likert scale from 0 (never) to 4 (very often). Possible scores vary between 0 and 56, where a score of 28 or more corresponds to problematic Internet use. To ensure that participants answered based on their gaming habits, they were asked to think only about gaming and not about their general Internet use.

#### 2.3.8 Psychological distress

Psychological distress was assessed with the “*Index de détresse psychologique de l’enquête Santé Québec-14*” (58–60). This questionnaire, used in epidemiological surveys, was validated with respondents aged 15 years and over (α = .90). It included 14 items divided into four subscales that assessed the presence of symptoms of depression, anxiety, irritability, and cognitive problems. Scores varied between 14 and 56. In order to determine an appropriate cutoff score for a high level of psychological distress, the authors of the most recent survey conducted by the “*Institut de la Satistique du Québec*” were consulted (61). Given the absence of a validated cutoff score, the *Institut de la Statistique du Québec* recommends the use of a continuous score, where a higher score indicates more psychological distress.

#### 2.3.9 Self-esteem

Self-esteem was assessed using Rosenberg’s Self-Esteem Scale (62) validated in French (63) and composed of 10 items (α varied from 0.70 and 0.88). Possible scores varied between 10 and 40. The variable was dichotomized to compare “very low/low” (10-30) to “average/high” self-esteem (31-40).

#### 2.3.10 Impulsivity

Impulsive traits were assessed with a version of Eysenck’s Impulsivity Scale (64), adapted by Vitaro, Ferland, Jacques & Ladouceur (65). The internal consistency for the original scales varies from 0.74 with preadolescent boys to 0.85 with young male adults. The adapted version used by Vitaro et al. (65) consists of five yes/no statements that had the highest factor loadings on the original scales. The maximum score for the adapted scale is 5.

#### 2.3.11 Reported diagnosed physical or mental health problems

The presence of diagnosed physical or mental health problems was documented with two questions asking to report the presence of a diagnosed problem (yes or no).

### 2.4 Data analysis

All 176 participants were included in the analysis. Considering the descriptive nature of the study, descriptive statistics were used to characterize both groups (ESp and nESp) in terms of demographic and academic characteristics, offline and online activities, and psychological characteristics. This included frequency distributions for categorical variables. To understand the differences between these groups, Student *T*-tests were used to conduct group comparison on continuous scores and Chi squared tests were conducted on categorical scores using SPSS (version 26). Considering the exploratory nature of this study, *p* levels were set at *p* = .05 (bilateral) for *T-*tests and at *p* = .05 for all Chi squared tests.

## 3 Results

### 3.1 ESp characteristics

ESp in our sample have little experience with ES (77.6% less than 12 months) and only 16.4% report an interest towards professional aspirations. They spend an average of 14.4 hours a week (*SD* = 15.0) on ES. ESp engage in a set of games that foster competition (e.g., *League of Legends, Rocket League, Fortnite*). These games, regardless of their genre, typically involve short duration rounds where participants compete against each other.

### 3.2 Sociodemographic and academic characteristics

The two groups do not differ significantly in age (16.4 years), mother tongue (94.0% French), ethnic background (79.1% Canadian), family configuration, or employment status (**Table 1**). However, a significantly higher proportion of ESp report being “single” (94.0%) than nESp (81.7%) [χ^2^(1) = 5.399, *p* = 0.020; Cramer’s V = 0.175]. Lastly, the groups do not differ significantly in average academic grades or time spent on studies.

**Table 1:** Comparison of ESp and nESp sociodemographic and academic characteristics.

	ESp^†^(*n* = 67)	nESp^‡^ (*n* = 109)	*t*/χ^2^
Sociodemographic			
**Age**	16.4 (SD = 0.7)	16.4 (*SD* = 0.8)	0.009
**Ethnic background**			
Canadian	79.1%	85.3%	1.134
Others	20.9%	14.7%	
**Mother tongue**			
French	94.0%	94.5%	0.017
Other Languages	6.0%	5.5%	
**Family configuration**			
Nuclear	64.2%	56.9%	0.918
Others	35.8%	43.1%	
**Relationship status**			
Single	94.0%	81.7%	5.399**
In a relationship	6.0%	18.3%	
**Employment status**			
Unemployed	59.7%	71.6%	2.641
Part-time	40.3%	28.4%	
Academic			
**Mean grade**			
70% +	79.1%	79.8%	0.03
**Hours studying/week**	4.8 (*SD* = 7.3)	5.0 (*SD* = 7.9)	0.018

^†^eSports players; ‡non-eSports players.

*p<.05, **p<.01, ***p<.001; Student *T*-tests were used for continuous measures and Chi-squared tests for dichotomous variables.

### 3.3 Offline and online activities

There are no significant differences between the two groups in terms of time spent on offline activities or other online activities besides gaming and ES (**Table 2**). However, ESp spend significantly more time gaming [*t* (107.272) = 2.128, *p* = 0.036]. Furthermore, their total time spent online is significantly higher than nESp [*t* (101.130) = 4.194, *p* < 0.001].

**Table 2:** Comparison of ESp and nESp offline and online activities.

	ESp^†^ (*n* = 67)	nESp^‡^ (*n* = 109)	*t*
**Time spent on offline activities** (hours/week)	10.0 (*SD* = 8.9)	12.8 (*SD* = 13.0)	-1.705
**Time spent on online activities** (hours/week)			
Gaming *(excluding eSports)*	20.7 (*SD* = 19.4)	14.9 (*SD* = 13.8)	2.128*
eSports *(excluding gaming)*	14.4 (*SD* = 15.1)	–	–
Online activities *(excluding gaming/eSports)*	14.8 (*SD* = 12.0)	16.5 (*SD* = 12.1)	-0.924
Total online activity time	49.9 (*SD* = 32.0)	31.5 (*SD* = 20.7)	4.194***
**Gaming Expenses** ($CAN)			
Electronic/computer equipment purchases	994.6 (*SD* = 1379.8)	691.9 (*SD* = 1120.2)	1.581
Video games	298.6 (*SD* = 380.1)	206.8 (*SD* = 254.9)	1.911
Membership	45.3 (*SD* = 57.1)	48.2 (*SD* = 69.6)	-0.289
Skins	97.5 (*SD* = 138.2)	36.6 (*SD* = 87.4)	3.210**
Loot boxes	28.3 (*SD* = 68.2)	24.6 (*SD* = 78.8)	0.310
Total expenses	1434.6 (*SD* = 1505.3)	1007.9 (*SD* = 1332.7)	1.946

^†^eSport players; ‡non-eSport players.

*p<.05, ** p<.01, ***p<.001; Student T-tests were used for group comparisons.

Aside from spending more money on skins [*t* (96.871) = 3.210, *p* = 0.002], money spent on electronic equipment, memberships, and loot boxes and the total amount of money spent on gaming expenses does not differ significantly between the two groups.

### 3.4 Gaming motivations

There are no significant differences between the two groups in terms of gaming motivations on any subscales (**Table 3**).

**Table 3:** Comparison of ESp and nESp gaming motivations.

	ESp^†^ (n = 67)	nESp^‡^ (n = 109)	*t*
**Gaming Motivations**			
Intrinsic	12.5 (*SD* = 5.1)	12.1 (*SD* =4.1)	0.633
Integrated	9.2 (*SD* = 5.6)	8.0 (*SD* = 4.8)	1.511
Identified	9.5 (*SD* = 5.5)	9.1 (*SD* = 4.5)	0.519
Introjected	6.8 (*SD* = 4.5)	5.7 (*SD* = 3.7)	1.560
External	9.3 (*SD* = 5.4)	9.3 (*SD* = 5.1)	-0.054
Amotivation	6.6 (*SD* = 4.2)	6.0 (*SD* = 3.8)	0.941

^†^eSport players; ‡non-eSport players.

*p<.05, **p<.01,***p<0.001; Student T-tests were used for group comparisons.

### 3.5 Perceived positive and negative consequences of gaming

In terms of positive consequences of gaming (**Table 4**), significantly more ESp (11.9%) report positive consequences on their physical health than nESp (2.8%) [χ^2^(1) = 5.813, *p* = 0.022, Cramer’s V = -0.0184]. ESp also tend to report more benefits on their family relationships than nESp (22.4% vs. 12.8%) [χ^2^(1) = 2.746, *p* = 0.097, Cramer’s V = 0.125]. No other significant differences were observed between the groups in terms of positive consequences. Overall, the most important perceived positive consequences of gaming for ESp are on their social relations (74.6%), their general motivation (44.8%), their mental health (41.8%) and their level of concentration (38.8%).

**Table 4:** Comparison of ESp and nESp perceived positive and negative consequences of gaming.

	Perceived positive consequences			Perceived negative consequences		
	ESp^†^ (*n* = 67)	nESp^‡^ (*n* = 109)	χ^2^	ESp^†^ (*n* = 67)	nESp^‡^ (*n* = 109)	χ^2^
**Study/work**	6.0%	5.5%	0.017	55.2%	32.1%	9.170**
**Social relations**	74.6%	66.1%	1.434	10.4%	11.9%	0.090
**Family relations**	22.4%	12.8%	2.746	25.4%	34.9%	1.739
**Mental health**	41.8%	36.7%	0.454	17.9%	16.5%	0.057
**Academic motivation**	17.9%	13.8%	0.550	16.4%	11.9%	0.711
**General motivation**	44.8%	33.0%	2.444	10.4%	11.9%	0.090
**Concentration**	38.8%	31.2%	1.070	13.4%	11.9%	0.086
**Physical health**	11.9%	2.8%	5.813*	11.9%	6.4%	0.096
**Leisure/sport activities**	17.9%	14.7%	0.324	35.8%	28.4%	1.052
**Level of energy**	17.9%	12.8%	0.846	17.9%	16.5%	0.057
**Sleep**	3.0%	4.6%	0.290	47.8%	36.7%	2.101

^†^eSport players; ^‡^non-eSport players.

*p<.05, **p<.01,***p<.001; Chi-squared tests were used for group comparisons.

As for negatives consequences associated with gaming, ESp (55.2%) report more consequences on their study/work than nESp (32.1%) [χ^2^(1) = 9.170, *p* = 0.002, Cramer’s V = 0.228]. No other significant differences were observed between the groups in terms of negative consequences. Overall, the most important perceived negative consequences of gaming for ESp are on their sleep (47.8%), their other leisure activities (35.8%) and their family relationships (25.4%).

### 3.6 Psychological characteristics

There were no significant differences between the two groups in terms of psychological distress, self-esteem, impulsivity, psychological or physical diagnosis (**Table 5**). However, significantly more ESp (32.8%) are considered as potentially problematic gamers than nESp (12.8%) [χ^2^ (1) = 10.193, *p* = 0.001, Cramer’s V = 0.241].

**Table 5:** Comparison of ESp and nESp psychological characteristics.

	ESp^†^ (n = 67)	nESp^‡^ (n = 109)	*t/*χ^2^
**Problematic gaming**	32.8%	12.8%	10.193**
**Psychological distress**	27.2 (*SD* = 7.5*)*	26.8 (*SD* = 9.0)	0.338
**Self-esteem** (very low/low)	58.2%	53.2%	0.419
**Impulsivity**	7.3 (*SD* = 1.2)	7.4 (SD = 1.2)	-0.518
**Diagnosed mental health problem**	41.8%	48.6%	0.780
**Diagnosed physical health problem**	19.4%	10.1%	3.055

^†^eSport players; ‡non-eSport players.

*p<.05, **p<.01,***p<0.001; Student T-tests were used for continuous measures and Chi-squared tests for dichotomous variables.

## 4 Discussion

Among the most recent school efforts to use technology to engage students, a growing number of high schools now offer ES as curricular or extracurricular activities. Considering the known consequences associated with excessive gaming and the absence of scientific documentation on adolescent ES players, the implementation of ES in high school settings raises many concerns about the potential risks and benefits associated with this activity. To address these concerns, the goal of this study is to draw an initial profile of adolescent ES players in a high school by comparing them to their classmates who also play video games but don’t engage in ES.

Our findings show that adolescent ES players in high school have limited experience in this activity and that most of them don’t plan to play professionally in the future. For the vast majority of these adolescents, ES seem to constitute a leisure activity in their school trajectory rather than a professional aspiration in their life. As a result, it is difficult to compare these adolescents to semi-professional or professional adults since their expectations towards this activity differ greatly (27, 28, 31, 34, 35, 66). Aside from a higher likelihood of being single, the sociodemographic and academic characteristics of ES players do not differ from their recreational gamer peers. This higher proportion of ES players who are single could be due to their elevated screen time spent online or gaming, which could lower opportunities to meet potential partners. However, the study design does not allow to support an explanation of whether ES adds a delay in the development of experiences or interferes with the development of skills required to establish healthy and satisfying romantic relationships.

Our findings also show that adolescent ES players report an elevated screen time. More specifically, ES players in our study spend an average of 14 hours weekly practicing ES, in addition to time playing videogame and other online activities. In comparison, adult ES players spend over 10 hours per day practicing ES, that is, an average of about 42 hours weekly (27, 28, 38). Although adolescent ES players in high schools spend much less time weekly on ES than professionals (27, 28), they still spend a non-negligible amount of time gaming and on other online activities. Overall, ES players spend significantly more time online (50 hrs/week) than their recreational gamer peers (32 hrs/week), which could potentially interfere with their schoolwork. This difference in ES players’ online time, also observed in another study (33), can mostly be explained by the specific time spent on ES in addition to the time spent gaming and on other online activities. Thus, time spent on ES does not appear to have replaced gaming habits already in place prior to the introduction of ES but is rather added to them. Therefore, it seems that the involvement in ES, without actively aiming at reducing the time invested in other online activities, cannot be seen as an avenue to reduce the overall screen time in adolescents. This cumulative effect should be considered in ES programs due to the known consequences associated with elevated screen time and the effect of a sedentary lifestyle (66–69).

Adolescent ES players’ gaming is also associated with more monetary spending, specifically to purchase skins. Considering the large amount of time that ES players spend gaming, these purchases could be explained by a desire for a certain prestige or social status that these items can provide within games (70). Furthermore, since adolescent ES players spend a considerable amount of money on gaming-associated expenses and that most do not have a job, their gaming expenses could be a potential risk to them. This could suggest that they may rely on their parents to pay or to support their gaming expenses, which could contribute to family conflicts regarding their gaming activities. Considering the presence of monetization schemes aimed at young players, prevention efforts should include the financial aspects associated with gaming to prevent adolescents from developing negative financial consequences (70, 71).

In terms of gaming motivations, ES players play video games for various reasons, however no group differences were observed. Since adult ES players are motivated by the desire to improve their skills, to compete, to socialize or to escape daily problems (31, 33, 41), this observation was surprising. This could be explained by the fact that ES players from this study seems to mostly engage “recreationally” in ES, and since they play similar popular competitive video game as their peers (e.g., *League of Legends, Fortnite, Rocket League*), adolescent ES players gaming motivations could be more similar to their peers than to adult professional gamers.

In terms of the perceived effects of gaming on their lives, adolescent ES players report both positive and negative consequences. More specifically, on one hand, ES players report positives consequences on their physical and mental health, on their social relationships, on their general motivation and on their concentration. In line with Freeman and Wohn (46), it appears that for adolescents, the social components of ES are very important. Those social benefits could potentially explain the perceived effect on their mental health and their general motivation. It is also interesting to note that 22.4% of ES players also reported positive consequences on their family relationships. This could potentially be explained by the structured and supervised aspect of the program, which could provide reassurance to parents who may worry about their child’s gaming habits. Participating in an ES program may also ease at home discussions about gaming, thus deconstructing parents’ negative perception about gaming, which could reduce tensions or conflicts associated with gaming among the family (51, 52). These results should be considered by schools since they highlight potential benefits that could be associated with ES programs (e.g., physical health, social relationships). On the other hand, adolescent ES players report negative consequences in terms of their education, their family relationships, their other leisure activities, and their sleep. Although their academic results and study time do not differ from their counterparts, the main perceived negative consequences reported by ES players are on their academic and professional lives. Also, almost half of the ES players reported that gaming had consequences on their sleep and on their other leisure activities. In opposition to those who perceived benefits, 25.4% of adolescent ES players reported negative consequence from gaming in their family relationships. This could highlight the fact that ES players are not a homogenous group, and that different profiles may exist – some who spend more time on gaming activities, spend more money on gaming related expenses and experience more harm – and others who have a more balanced involvement in ES and experience more benefits.

An important result of this study is that ES players differ significantly in terms of potential gaming disorder in comparison to their gamer peers. Indeed, our findings show that 2.5 times more ES players are considered potential problematic gamers compared to non-ES players. This difference is also observed in other studies comparing adults ES players (33, 40, 41) and could suggest that ES players present a greater risk of developing a gaming disorder than recreational gamers. A recent study conducted on 123 262 young adult gamers highlighted that gaming disorder symptoms are associated with gaming 35 hours per week or more (72). Consequently, the cumulative time that ES spend gaming could put them at risk. Furthermore, considering that Montag et al. (41), highlighted that aspiring professionals may be more at risk than recreational or professional gamers, adolescents aspiring to become professional may be at a higher risk of developing problematic gaming than adolescents for whom ES remains a recreational activity.

In terms of their mental health, contrary to what was observed in the scientific documentation (37, 51), adolescent ES players do not differ from their classmates in their levels of impulsivity, self-esteem, or psychological distress. These results are surprising because these variables are known risk factors in the development of gaming disorder in adolescents (51, 52, 73) This difference could be explained by the fact that our sample is composed of high school students, who live with their parents, are single, and don’t have jobs. With few of the responsibilities associated with adult life, it is possible that adolescents’ high level of involvement in gaming has not yet led to a significant level of psychological distress which is observed in older ES players (27, 28).

Finally, the study conducted by Trotter et al. (29) focused mostly on self-regulation, positive youth development and growth mindset, hence the results from the present study, to our knowledge, are the first to draw a comprehensive profile of adolescent ES players in a school context. Considering the already existing concerns surrounding ES and that ES research is still in its infancy, the present exploratory design seems appropriated. However, future studies would benefit from relying on designs including hypotheses and targeted analysis to confirm the results obtained. While interesting, the results from the present study must be interpreted in light of its limitations. Firstly, the cross-sectional exploratory design of the study does not allow causal links to be made between ES and gaming disorder. Secondly, the limited number of participants in each group could also have affected the statistical power, making it more difficult to detect differences between the groups. Future research should aim to conduct *a priori* power analysis to ensure they have sufficient statistical power. Thirdly, longitudinal studies documenting risk factors that existed before the enrolment in this activity are needed to better understand ES players’ trajectories and the effect of this activity on their live. Finally, since our sample was mainly composed of males, our findings cannot be generalized to the female population.

## 5 Conclusion

In summary, although they are similar in many ways, adolescents involved in ES as an extracurricular activity spend much more time online than their peers. Adolescent ES players perceive both, positive and negative consequences from gaming. On one hand, they report positive consequence on their physical and mental health, social and family relations, and on their motivation. On the other hand, they report negative consequences on their work or education, on their other leisure activities, on their family relations and on their sleep. Considering the proportion of ES players being potentially problematic gamers, specific prevention efforts must be implemented in ES programs. Prevention efforts should aim to raise adolescents’ awareness on the total time they spend on their online activities (74, 75). It is recommended that prevention efforts adopt a nuanced point of view and address simultaneously positive and negative consequences of gaming, while touching various spheres of life such as family relationships as well as the balance between online and offline activities. Furthermore, school counselors should be equipped to detect gaming problems. Early detection and intervention can help prevent the onset of problems and the negative effects on their educational trajectory. Finally, further studies are needed to document the profile and trajectory of ES among young people. Only in this way can we better understand the long-term impact of this type of activity in high schools.

## Data availability statement

The datasets presented in this article are not readily available because of the specificity of the sample. Requests to access the datasets should be directed to Magali Dufour (dufour.magali@uqam.ca).

## Ethics statement

The studies involving humans were approved by the “Comité Institutionnel d’Éthique de la Recherche avec des Êtres Humains (CIEREH)” from the “Université du Québec à Montréal”. The studies were conducted in accordance with the local legislation and institutional requirements. Written informed consent for participation in this study was provided by the participants’ legal guardians/next of kin.

## Author contributions

AL: Conceptualization, Data curation, Formal analysis, Investigation, Methodology, Project administration, Resources, Writing – original draft, Writing – review & editing. MD: Conceptualization, Data curation, Formal analysis, Methodology, Supervision, Writing – review & editing. MG: Conceptualization, Data curation, Formal analysis, Methodology, Supervision, Writing – review & editing. DJ: Methodology, Validation, Writing – review & editing.
